# Temporal dynamics and intra-farm variability of animal welfare indicators in fattening pigs

**DOI:** 10.3389/fvets.2026.1797211

**Published:** 2026-03-30

**Authors:** Cecilia Aguilar-Vega, Antonio Velarde, Joaquim Segalés, Diego Pérez, Marc Bagaria, Beatriz Garcia-Morante

**Affiliations:** 1 IRTA, Animal Health, Centre de Recerca en Sanitat Animal (CReSA), Campus de la Universitat Autònoma de Barcelona (UAB), Bellaterra, Catalonia, Spain; 2 Unitat mixta d’investigació IRTA-UAB en Sanitat Animal, Centre de Recerca en Sanitat Animal (CReSA), Campus de la Universitat Autònoma de Barcelona (UAB), Bellaterra, Catalonia, Spain; 3 World Organization for Animal Health (WOAH) Collaborating Centre for the Research and Control of Emerging and Re-Emerging Swine Diseases in Europe (IRTA-CReSA), Bellaterra, Catalonia, Spain; 4 IRTA, Animal Welfare, Veïnat de Sies, Monells, Catalonia, Spain; 5 Departament de Sanitat i Anatomia Animals, Facultat de Veterinària, Universitat Autònoma de Barcelona (UAB), Campus de la UAB, Bellaterra, Catalonia, Spain

**Keywords:** animal welfare assessment, pig, reliability, temporal dynamics, Welfare Quality^®^ protocol

## Abstract

The Welfare Quality^®^ (WQ^®^) protocols are broadly implemented for monitoring farm animal welfare and certification purposes. However, they represent a single snapshot in time, demand a significant amount of time for execution, appraise only a subset of the animals, and to achieve high repeatability among assessors, they must be continuously trained. In growing pigs, the dynamic nature of physiological and behavioural changes during the production cycle may affect the consistency of monitoring over time and thus the reliability of the method. Hence, the aim of the present study was to evaluate the temporal variability of principles, criteria and measures of the WQ^®^ protocol in growing pigs, and the intra-farm (between buildings) variability of selected measures. The full WQ^®^ protocol was applied every two weeks during the fattening period in two separate buildings of the same farm by the same two trained assessors (seven and eight visits respectively). A descriptive temporal analysis was conducted, and the relation of measures was explored using Spearman’s correlation. Intra-farm variability of selected measures was assessed for each visit using a permutation test for independence. Certain variability in some measures, criteria and principles was observed depending on the timing of the assessment during the growing period. The “good housing” principle was the most fluctuating variable over time, due to the substantial downward trend of the scores of both the “comfort around resting” and “ease of movement” criteria. Although the “good health” principle was the most stable in terms of absolute score, the categorization changed in one assessment in one of the studied buildings. Both “good feeding” and “appropriate behaviour” remained stable in their categorization. More variability was observed at the criteria and measure levels. In terms of intra-farm variability, measures from four conditions were significantly different, in at least one visit, being the “fear of humans” measure the most frequently variable. This study highlighted the possible differences that may arise in WQ^®^ scoring depending on the time of the assessment during a growing period. The temporal dynamics and intra-farm variability of this protocol outcomes warrant further investigation to improve the reliability of welfare monitoring protocols.

## 1 Introduction

Animal welfare refers to “the physical and mental state of an animal in relation to the conditions in which it lives and dies” (1). The European Union acknowledges the importance of farm animal welfare, but efforts to improve its protection are still needed (2). Traditionally, good animal welfare was based in the “five freedoms” states developed by the former Farm Animal Welfare Council (FAWC) (3). However, this approach has evolved into the more comprehensive “five domains” model (4), which not only considers the absence of negative states but also emphasizes the promotion of positive experiences. Under this model, animal welfare is evaluated across five key domains: (i) nutrition, preventing from prolonged hunger and thirst; (ii) environment, addressing comfort and suitable living conditions; (iii) health, safeguarding against pain, injury, or disease; (iv) behaviour, allowing the expression of natural behaviours; and (v) mental state, encompassing emotional well-being influenced by the other four domains (4).

The Welfare Quality^®^ (WQ^®^) protocol emerged, based on the “five freedoms” framework, to provide a standardized estimate of the farm’s welfare for different species (5). This protocol is widely used in the pig sector to assess and certify animal welfare. It tackles different aspects of the farm facilities as well as the physical and emotional state of the animals. The overall assessment of the farm’s welfare is based on four principles: “good feeding”, “good housing”, “good health”, and “appropriate behaviour”. Each principle encompasses several criteria, whose scores are calculated based on a different number of related measures that are rated during the farm’s visit. The farm can be then categorized regarding animal welfare in descending order as “excellent”, “enhanced”, “acceptable” or “not classified”, based on the aggregating scoring of the measure for the four principles (5).

Reliability, along with validity and feasibility, has been identified as a crucial aspect in the assessment of animal welfare (6, 7). Reliability refers to the reproducibility of a measurement (8, 9). Temple et al. (2013) distinguished three different sources of variability over time: (i) unpredictable time-associated variability, (ii) minor routine modifications, maintaining the management and resources of the farm, and (iii) methodological changes (10). Some studies on fattening pigs have compared test–retest reliability of several animal-based measures on a year interval (10) or have compared same age groups of different batches (11, 12). Recent research has examined the test–retest reliability of twenty one animal-based measures from the WQ^®^ protocol and ten from other sources on the same animals throughout the rearing period (13). However, the study’s sampling strategy differs from the cross-sectional approach typically used in field applications of the WQ^®^ protocol (5). Friedrich et al. (2019a, 2019b) evaluated the consistency over time of the full WQ^®^ protocol for a different rearing stage (sows and piglets) (14, 15). Other authors have reported age related effects of several animal-based measures in fattening pigs, although lacking consecutive visits of the farms (16). Therefore, a knowledge gap still exists regarding the temporality and variability over time of animal welfare parameters in fattening pigs assessed using the full WQ^®^ protocol throughout a single fattening period.

To account for intra-farm variability, the WQ^®^ protocol requires animal welfare measures to be assessed on a representative sample. Therefore, when pigs are housed across multiple buildings, it is necessary to include a selection of pigs housed in different buildings in the evaluation to ensure a thorough and accurate assessment (5). Supporting this approach, some studies have highlighted differences in certain animal-based welfare measures between housing units in the same farm, such as pens (17), or buildings with different ventilation system (18).

In the current study, we investigated the temporal variability of animal welfare scores during the fattening period, as well as intra-farm variability. We hypothesised that physical, behavioural, and physiological changes occurring in pigs throughout the fattening period influence the scores of certain welfare measures, reflecting actual changes in welfare conditions rather than inconsistencies in scoring. Moreover, we anticipated that animals within the same farm would exhibit similar scores. Thus, the foremost aim of this study was to evaluate the temporal and intra-farm variability over time of certain measures of the WQ^®^ protocol in a single batch of fattening pigs located in two different buildings.

## 2 Materials and methods

### 2.1 Study farm

**Figure 1:**
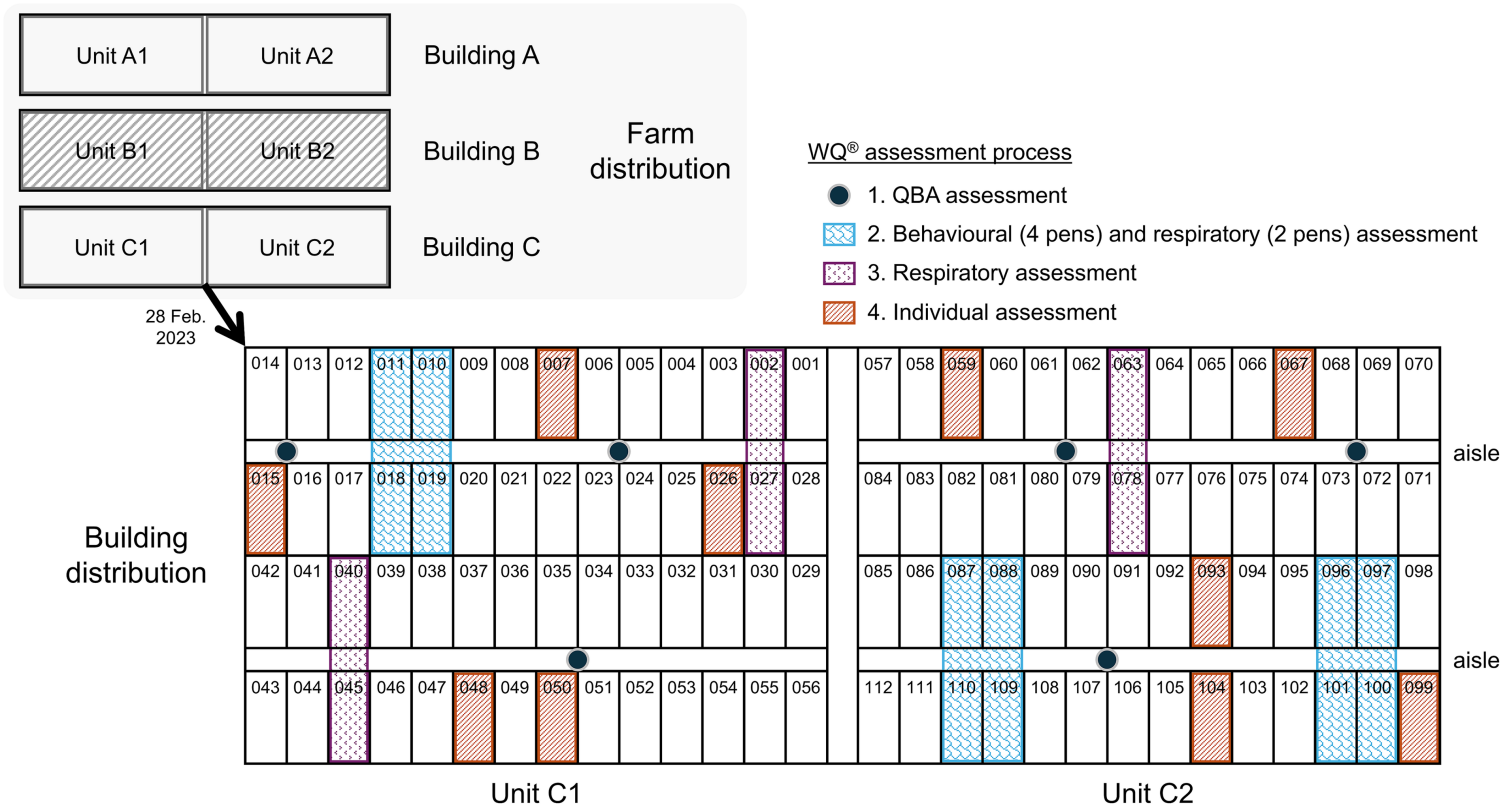
Graphical representation of the distribution of the buildings, units, and pens of the study farm (the dashed building was not included in the WQ^®^ assessment). The figure also illustrates the implementation of the WQ^®^ protocol during the assessment of the animals in building C conducted during the second visit (28 February 2023). QBA, quality behaviour assessment.

Data was obtained from a commercial fattening farm located in the province of Lleida (Northeastern Spain). It consisted of three separate buildings, each with two barns (units) connected by a central corridor (Figure 1). The three buildings were aligned in parallel and shared an identical architectural design. All units operated under an all-in/all-out management system, each comprising between 680 and 694 pigs at the beginning of fattening (a total of 4,120 pigs in the farm). Each unit contained 56 pens, each measuring 9 m^2^ and housing 12 to 14 fattening pigs, with males and females mixed, except in the hospital pens. All pigs had been previously tail-docked, and none were castrated. Pens had fully slatted concrete floors; floor space was 0.7 m^2^/pig approximately and there was one feeder and drinker per pen. Pigs were fed *ad libitum* a standard cereal-soy diet and water was provided by stainless steel pig automatic drinking bowls with nipples. Straw was provided in each pen as an environmental enrichment material via a dispenser. During the period of study, no major management changes occurred on the farm, such as changes in the vaccination plan, resource provision, or caretakers. Environmental temperature inside each building was similar throughout the fattening period. The mean daily temperature was 26.03 °C (range: 22.3 °C to 30.8 °C) in building A and 25.85 °C (range: 17.9 °C to 31 °C) in building C. Humidity was more variable between buildings, with a mean daily relative humidity of 62.68% (range: 51 to 71%) in building A and 66.15% (range: 32 to 76%) in building C.

### 2.2 Animal welfare assessments

From February to May 2023, eight welfare assessments following the WQ^®^ protocol for growing pigs (5) were carried out on the same day every two weeks approximately, on two out of the three buildings of the farm (A and C; Figure 1), when possible. The first visit was performed on 9 February 2023 for both buildings. The timing of the last visit varied between buildings, being on 12 May 2023 for building C, and on 26 May 2023 for building A. Building C was not evaluated during the last visit due to an insufficient number of animals, as a proportion had already been sent to the slaughterhouse upon reaching the target finishing weight. Hence, building A was evaluated eight times, while building C, seven times. All measurements were obtained consecutively through a fattening period of a unique batch of animals, with pigs ranging from 23.5 to 110 kg of body weight. Furthermore, each assessment was performed by the same two assessors, both of whom received the same training. Throughout the assessment process, each assessor consistently evaluated the same unit across buildings—one assigned to Unit A1 and C1 and the other to Unit A2 and C2.

### 2.3 The Welfare Quality^®^ protocol for growing pigs

**Figure 2:**
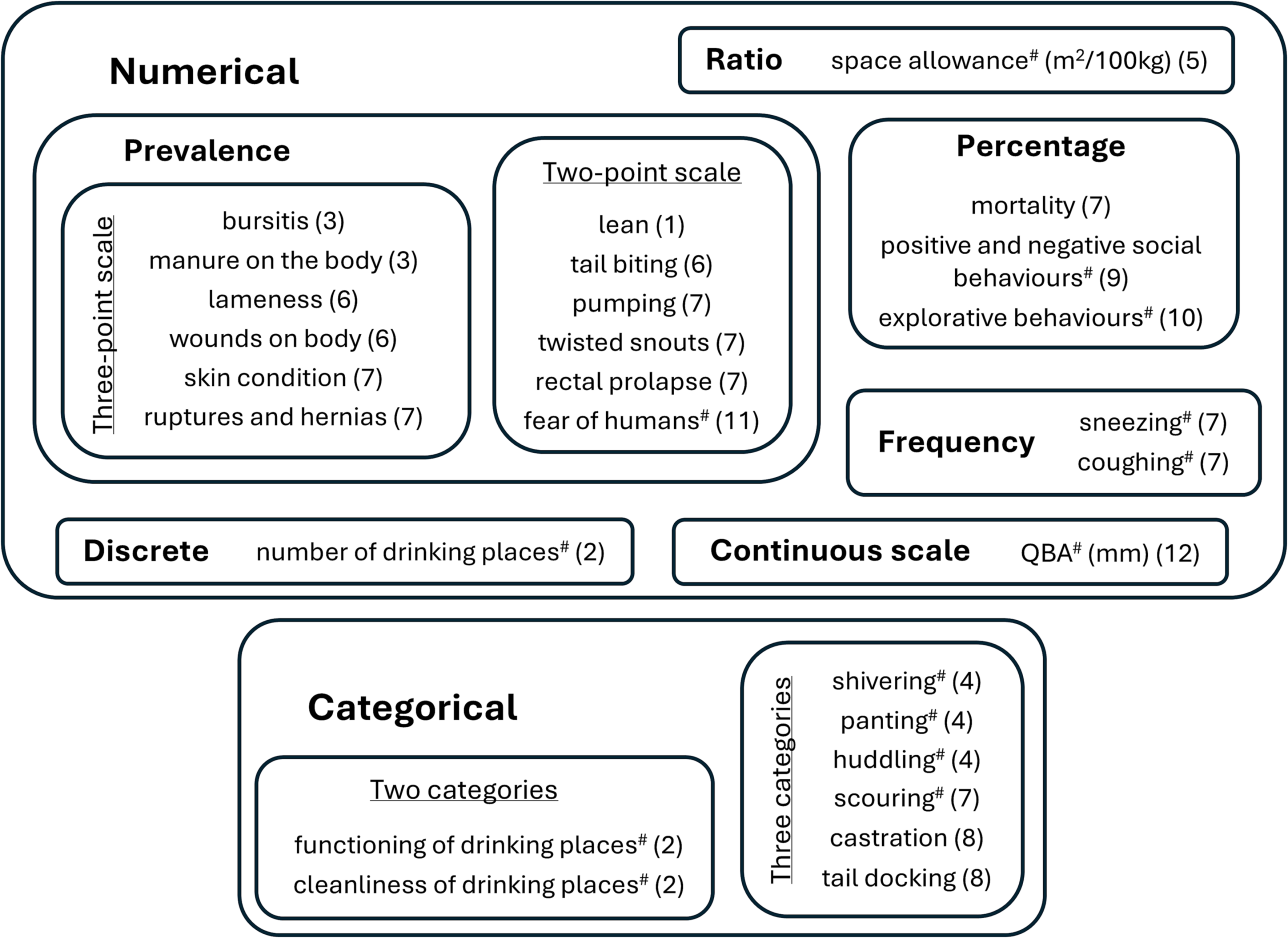
Characteristics of Welfare Quality^®^ measures. The welfare criteria listed in Table 1 to which measure belong are in parentheses, and measures obtained at pen-level are tagged (#); the others are collected at individual level but are subsequently aggregated to generate a pen-level score. QBA, quality behaviour assessment.

The overall assessment of the farm’s welfare is based on four principles (good feeding, good housing, good health, and appropriate behaviour) and 12 criteria (absence of prolonged hunger, absence of prolonged thirst, comfort around resting, thermal comfort, ease of movement, absence of injuries, absence of disease, absence of pain induced by management procedures, expression of social behaviours, expression of other behaviours, good human-animal relationship, and positive emotional state), which are calculated based on 49 different related measures taken during the farm’s visit (Table 1; Figure 2). Scores for both criteria and principles were calculated using the standardized computational procedures described in the Welfare Quality^®^ protocol (5), with no study-specific modifications. Criteria and principles are scored on a scale from 0 to 100, with 0 representing the worst and 100 the best possible scenarios. Four welfare categories are defined: (i) “not classified” (0–20) indicates low animal welfare, representing an unacceptable situation; (ii) “acceptable” (>20–55) shows that welfare meets minimum standards or slightly exceeds them, but is not considered good; (iii) “enhanced” (>55–80) considers that animal welfare is good; and “excellent” (>80–100) represents the highest level of animal welfare. Criteria scores rely on three main types of mathematical calculations: (i) assigning scores based on decision trees, (ii) weighted sums of measures expressed as percentages, and (iii) the application of predefined alarm and warning thresholds (5). On the other hand, measures provide non-aggregated values to welfare-related aspects of the animals in the farm. They can be (i) animal-based, which are drawn by visual inspection of some animals during the farm visit, (ii) management-based, that are asked to the farm manager and relate to procedures applied to animals or, (iii) resource-based, that are related to the facilities as well as the environment, and are measured by the assessor (5). Measures can be numerical or categorical, have different units and are obtained from the individuals or at the pen level (Figure 2).

**Table 1:** Description of principles, criteria and measures of the Welfare Quality^®^ protocol applied to growing pigs and finishing pigs (5).

Welfare principles	Welfare criteria	Measures	Calculation of criteria scores
Good feeding	1. Absence of prolonged hunger	Body condition score	I-spline functions from index
	2. Absence of prolonged thirst	Water supply^1^: number of drinking places, functioning and cleanliness of drinkers	Decision tree
Good housing	3. Comfort around resting	Bursitis, absence of manure on the body	Choquet integral from scores obtained by I-spline functions from index
	4. Thermal comfort	Shivering, panting, huddling	Decision tree
	5. Ease of movement	Space allowance^1^^,^^2^	I-spline functions from index
Good health	6. Absence of injuries	Lameness, wounds on the body, tail biting	Choquet integral from scores obtained by I-spline functions from index
	7. Absence of disease	Mortality^2^, coughing, sneezing, pumping, twisted snouts, rectal prolapse, scouring, skin condition, ruptures and hernias	I-spline functions from index calculated using warning and alarm thresholds
		8. Absence of pain induced by management procedures		Castration^2^, tail docking^2^	Decision tree
	Appropriate behaviour	9. Expression of social behaviours	Social behaviours (positive and negative)	I-spline functions from index
10. Expression of other behaviours		Exploratory behaviours	I-spline functions from index
11. Good human-animal relationship		Fear of humans	I-spline functions from index
12. Positive emotional state		Qualitative behaviour assessment (QBA), based on 20 emotional states	I-spline functions from index (obtained by weighted sum)

1 Resource-based measure.

2 Management-based measure.

### 2.4 Sampling strategy and welfare assessment procedures

The WQ^®^ protocol prescribes a structured sampling strategy to ensure representative welfare assessments of growing pigs on farms (5). In every visit the assessors followed an identical approach protocol that is described below (5). At the beginning of the assessment, management-based measures were collected through an interview with the animal unit manager. During each visit, pens were randomly selected prior to the assessors’ entrance into the facilities to ensure coverage of all spatial areas within the housing unit (e.g., near doors, in the middle, and at the back of rooms), thereby minimizing location bias and constituting a representative sample (Figure 1). Consequently, different animals from the batch were assessed during each visit. In accordance with the protocol, qualitative behaviours assessment (QBA) was performed first at six observation points, with a total observation time not exceeding 20 min. Afterwards, respiratory disorders (coughing and sneezing) were assessed also at six observation points, each covering two pens (25–28 pigs), for 5 minutes. For three of these points, assessments were conducted during the five-minute acclimation period preceding behavioural observations. Social and exploratory behaviours were then assessed at three observation points, each covering four pens (48–56 pigs), using five scan samples at two-minute intervals. Lastly, measures of thermal comfort, fear-of-humans, and health- and resource-based assessments were evaluated in the same 10 pens per building. Panting, shivering, and huddling were recorded from outside the pens prior to entry to avoid disturbing the animals. Upon entering each pen, the fear-of-humans test was conducted first, followed by health- and resource-based measurements. These included evaluations of body condition, bursitis, manure on the body, wounds, tail biting, lameness, respiratory signs (pumping, twisted snouts), rectal prolapse, scouring, skin condition, ruptures/hernias, water supply, and space allowance. Figure 1 illustrates an example of sequence, sample size, and location of the recorded measures conducted in a building during a single visit.

### 2.5 Statistical analyses

Intra-farm variability was assessed by comparing the values obtained for selected measures from the two buildings under evaluation at the pen level during each visit. Only measures recorded by assessors during the respiratory and individual assessments of the WQ^®^ protocol were included, while variables with fewer sampling sites and overall prevalence below 3% were excluded. For respiratory measures, the frequency of coughing or sneezing per 5-min period for each evaluated pen was used. For other individual continuous measures, the percentage of animals in the pen exhibiting the condition was considered, whereas for categorical measures, the assigned pen category was used. Because pens in both buildings were randomly selected according to the WQ^®^ protocol, and both buildings belong to the same farm, they could not be considered paired nor fully independent. Therefore, a permutation test of independence was applied to both continuous and categorical measures using the R package “coin” (19), rather than traditional hypothesis-testing methods based on distributional assumptions –parametric or rank-based tests. Differences between buildings were considered statistically significant when *p*-value < 0.05. Furthermore, to explore possible associations between measures, we calculated a Spearman’s rank correlation using the aggregated value of each measure at the visit level. Measures with low prevalence or minimal variation over time were excluded from the analysis. Only significant correlations were analysed (*p*-value < 0.05), and correlations superior to 0.7 were considered high. Additionally, the weight of the animals was included as a time-dependent variable to capture changes over time. Data analysis and visualization were performed in R v.4.5.2 (20) using the following packages: “tidyverse” (21), “Hmisc” (22), “ggcorrplot” (23), and “gridExtra” (24).

## 3 Results

The period between visits was 15.14 days (± 2.54 days), with a minimum of 11 days and a maximum of 19 days between visits. In accordance with the aggregation process of the WQ^®^ protocol, results are presented in a hierarchical structure, beginning with measures, followed by criteria, and concluding with principles.

### 3.1 Measures

Descriptive statistics for quantitative measures with some variation over time are gathered in Table 2, and intra-farm correlation for certain variables in Table 3. Concerning the measures related to the principle of good feeding, no lean animals were observed during any of the assessments. Drinkers functioned properly in every visit; however, they were only deemed clean in the first two assessments for building A, and the first and third for building C.

**Table 2:** Descriptive statistics of measures scores of the Welfare Quality^®^ protocol for buildings A and C with their variability.

Criteria	Measures (unit)	Building A				Building C			
		Mean^1^	sd	Range	IQR	Mean^1^	sd	Range	IQR
-	Number/pen	12.20	1.19	[9.40, 12.90]	0.58	12.61	0.42	[11.80, 13]	0.45
-	Weight (kg)	80.60	29.09	[32.8, 113.2]	41.35	75.94	28.02	[32.80, 110.10]	36.95
3	Bursitis 0 (%)	68.28	17.72	[43.62, 96.12]	22.33	69.60	17	[51.18, 93.94]	27.47
3	Bursitis 1 (%)	22.54	10.28	[3.88, 35.11]	9.35	22.23	10.11	[6.06, 35.20]	12.99
3	Bursitis 2 (%)	9.18	9.06	[0, 22.03]	14.08	8.17	7.25	[0, 17.32]	12.63
3	Manure 0 (%)	70.68	10.12	[60.32, 89.84]	9.54	67.64	20.75	[44, 98.48]	30.12
3	Manure 1 (%)	24.21	10.06	[5.47, 33.33]	7.64	24.39	14.01	[1.52, 38.40]	19.16
3	Manure 2 (%)	5.10	2.35	[1.06, 8.06]	2.74	7.96	7.13	[0, 17.60]	11.68
5	Space allowance (m^2^/100 kg)	1.05	0.48	[0.67, 2.10]	0.32	1.08	0.51	[0.68, 2.10]	0.43
6	Lameness 1 (%)	1.04	0.82	[0, 2.34]	1.05	0.78	0.63	[0, 1.57]	0.77
6	Lameness 2 (%)	0.11	0.3	[0, 0.85]	0	0	0	[0, 0]	0
6	Wounds 1 (%)	16.67	5.79	[7.75, 23.40]	9.43	17.40	6.01	[9.85, 26.40]	8.65
6	Wounds 2 (%)	1.16	1.25	[0, 3.39]	1.80	1.56	1.02	[0.76, 3.20]	1.54
7	Cough frequency (per animal during 5 min)	0.40	0.36	[0.10, 1.15]	0.44	0.29	0.27	[0.03, 0.83]	0.24
7	Sneeze frequency (per animal during 5 min)	0.12	0.09	[0.02, 0.26]	0.10	0.16	0.09	[0.04, 0.32]	0.10
7	Pumping (%)	0.29	0.83	[0, 2.34]	0	0	0	[0, 0]	0
7	Skin condition (%)	0.21	0.38	[0, 0.85]	0.20	0.45	0.63	[0, 1.60]	0.78
7	Hernia 1 (%)	0.72	0.66	[0, 1.56]	1.19	1.11	0.89	[0, 2.36]	1.18
7	Hernia 2 (%)	0	0	[0, 0]	0	0.11	0.29	[0, 0.77]	0
7	Mortality (%)	2.89	1.41	[1.77, 5.14]	0.84	3.05	1.44	[1.77, 5.14]	1.41
9	Social behaviour (%)	10.03	2.61	[7.06, 14.54]	3.89	12.16	4.66	[7.79, 20.96]	5.18
9	Negative social behaviour (%)	3.26	1.07	[2.14, 5.18]	1.08	3.57	1.04	[1.96, 4.97]	1.24
10	Exploration of the pen (%)	35.58	9.42	[18.05, 43.01]	13.29	38.94	7.05	[28.29, 47.19]	9.97
10	Exploration of enrichment material (%)	13.53	8.21	[0.41, 24.03]	11.25	16.40	7.66	[6.38, 27.77]	9.89
11	Fear of humans (%)	67.50	17.53	[40, 90]	17.50	25.71	19.02	[0, 50]	30
12	Active (mm)	5.60	1.82	[1.70, 7.30]	1.28	4.56	2.10	[1, 7]	2.50
12	Relaxed (mm)	5.81	2.13	[3, 8.50]	2.98	8.19	1.57	[5.80, 11]	0.95
12	Fearful (mm)	3.90	1.77	[1, 6.70]	1.53	2.10	1.03	[0.90, 3.40]	1.75
12	Agitated (mm)	3.78	1.63	[1.90, 6.60]	2.13	2.30	1.26	[1.20, 4.50]	1.65
12	Calm (mm)	6.51	1.88	[3.50, 10.10]	0.98	8.51	2.28	[5.30, 11]	3.15
12	Content (mm)	4.93	1.35	[3.50, 7.30]	1.98	5.94	1.45	[3.20, 7.30]	1.40
12	Happy (mm)	5.23	1.77	[2.40, 7.10]	2.53	5.53	1.99	[1.40, 7.10]	1.70
12	Tense (mm)	2.73	1.69	[0.80, 6]	2	2.01	1.08	[0.80, 4]	1.15
12	Enjoying (mm)	3.49	1.24	[1.50, 5.20]	1.55	4.34	1.89	[1.50, 6.60]	2.05
12	Frustrated (mm)	3.58	1.32	[1.50, 5.20]	1.85	3.30	2.05	[0, 6.30]	1.90
12	Sociable (mm)	5.74	0.79	[4.40, 6.90]	0.80	6.17	1.50	[4, 8.60]	1.60
12	Bored (mm)	5.40	2.05	[2, 7.90]	2.68	5.74	3	[2.20, 9]	5.35
12	Playful (mm)	4.53	2.29	[1.20, 8.20]	2.45	3.64	1.85	[1.20, 6.40]	2.20
12	Distressed (mm)	1.29	0.89	[0.20, 3.10]	0.70	1	0.65	[0.40, 2.30]	0.50
12	Positively occupied (mm)	4.78	1.41	[2.50, 6.90]	1.70	5.33	1.88	[3, 8.30]	2.50
12	Listless (mm)	2.33	1.17	[0.50, 4.10]	1.45	2.14	1.77	[0.60, 5.50]	1.80
12	Lively (mm)	5.33	1.39	[3.90, 7.80]	1.93	5.67	0.77	[4.60, 6.50]	1.25
12	Indifferent (mm)	4.09	1.77	[1.30, 6.50]	2.15	4.57	2.96	[0.40, 9.30]	3.45
12	Irritable (mm)	1.65	0.83	[0.30, 3.10]	0.63	1.33	0.50	[0.50, 2]	0.45
12	Aimless (mm)	5.08	1.30	[3, 6.70]	1.75	4.46	2.37	[0.90, 6.80]	3.75

Criteria: (1) Absence of prolonged hunger, (2) Absence of prolonged thirst, (3) Comfort around resting, (4) Thermal comfort, (5) Ease of movement, (6) Absence of injuries, (7) Absence of disease, (8) Absence of pain induced by management procedures, (9) Expression of social behaviours, (10) Expression of other behaviours, (11) Good human-animal relationship, (12) Positive emotional state. The number in some measures corresponds to the category of the severity of the condition: 0 = absence, 1 = moderate, and 2 = severe. sd, standard deviation; IQR, interquartile range.

1 Means for each building were calculated as the average of visit-level means (Building A: *n* = 8 visits; Building C: *n* = 7 visits). Each visit-level mean was based on pens assessed during that visit according to the WQ^®^ protocol.

**Table 3:** Results of the permutation test of independence (Z-statistics) and their significance for Welfare Quality^®^ protocol measures between buildings A and C across visits.

Variable	Visit						
	1	2	3	4	5	6	7
Cough frequency	1.60	0.34	−1.72	0.17	1.62	−0.82	0.46
Sneeze frequency	−0.98	1.46	0.40	−1.26	−0.49	−0.43	−1.07
Bursitis 0	0.75	2.73^a^	−2.45^a^	1.46	−0.38	0.79	0.05
Bursitis 1	−0.75	−2.37^a^	2.19^a^	−1.16	1.70	−1.94	−1.86
Bursitis 2	-	−1.51	1.37	−1.51	−1.20	0.14	0.51
Manure 0	−1.46	0.21	−0.93	0.74	0.08	1.65	2.23^a^
Manure 1	1.43	−1.27	0.98	−0.53	0.65	−0.84	−1.67
Manure 2	1.45	1.15	0.58	−0.82	−0.72	−1.48	−1.69
Wounds 0	0.80	1	−1.96	1.23	0.18	1.16	1
Wounds 1	−0.59	−0.55	2.14^a^	−1.01	−0.64	−0.99	−1.72
Fear of humans	3.62	7.13^a^	0.79	10.23^a^	12.16^a^	0.76	0

The number in some measures corresponds to the category of the severity of the condition: 0 = absence, 1 = moderate, and 2 = severe.

a *p*-value < 0.05.

**Figure 3:**
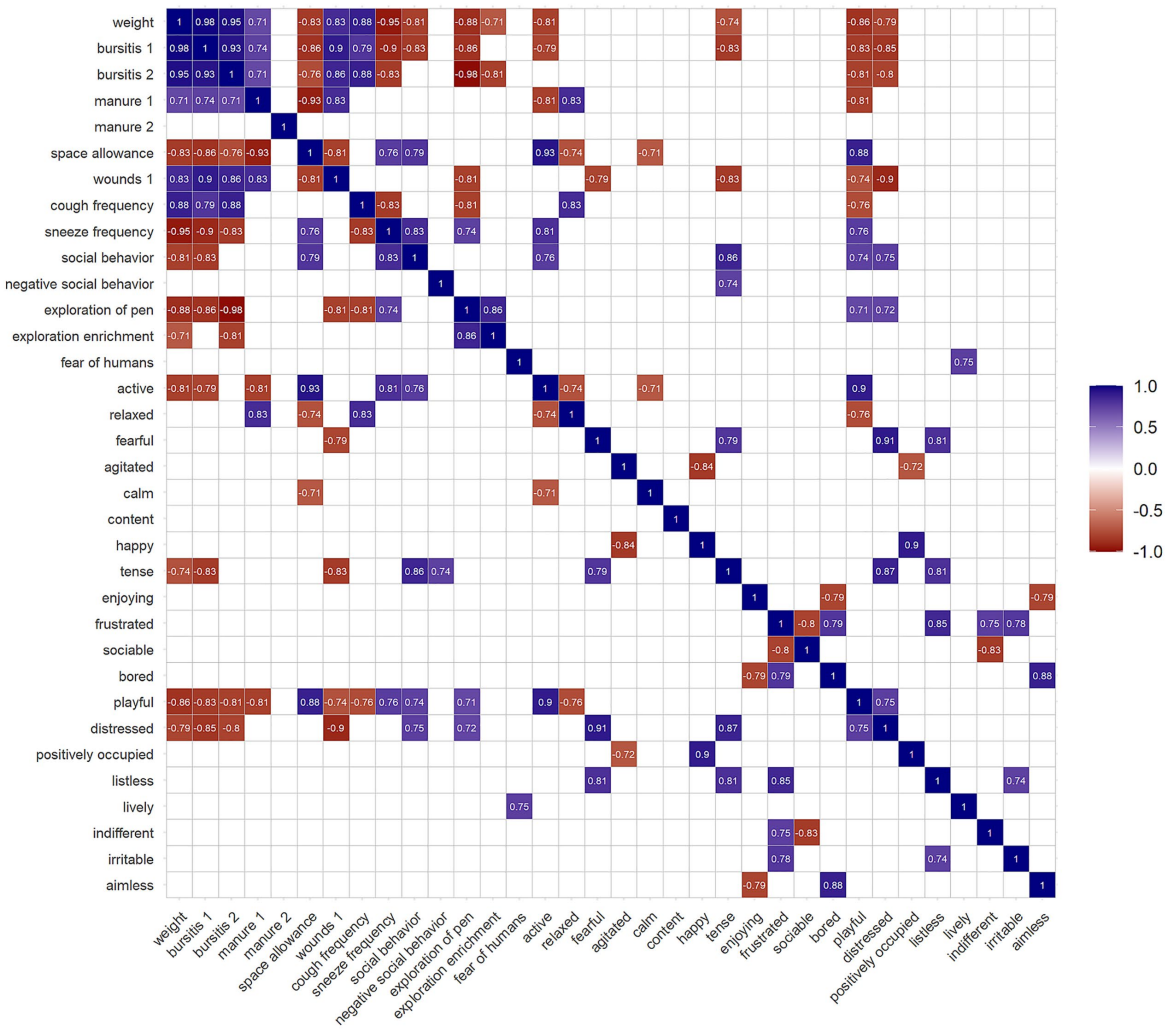
Significant correlation plot of selected measures from the Welfare Quality^®^ protocol. Blue represents positive correlation, red negative correlation, and blank spaces with no assigned value non-significant correlations (*p*-value > 0.05). The number in some measures corresponds to the category of the severity of the condition: 1 = moderate, and 2 = severe.

In terms of the principle of “good housing”, indicators of thermal stress such as shivering, panting, or huddling were absent. Moderate manure on the body and bursitis were the most prevalent conditions (Table 2). Although severe cases of these criteria were not frequently observed, both conditions became more prevalent in latter assessments, except for severe manure on the body in building A (Supplementary Figure 1). This is reflected in the strong correlation between both categories of bursitis and weight, as well as between moderate manure and weight (Figure 3). Additionally, absence of bursitis and moderate bursitis differed significantly between buildings in two of the visits, and the absence of manure on the body in one (Table 3). Space allowance showed a strong negative correlation with animal weight. It was also negatively correlated with moderate and severe bursitis, moderate wounds on the body, as well as moderate manure. Conversely, space allowance was positively correlated with sneeze frequency, social behaviour and some QBA measures (Figure 3).

Regarding some other measures related to animal health, no tail biting, twisted snouts nor rectal prolapse were observed in any building across all visits. Concerning scouring, some liquid manure (group category 1) was observed during the first assessment in building A. Within the “absence of injuries” criterion, moderate wounds on the body were more prevalent (Table 2), and statistically different in one of the visits (Table 3). Lame animals were infrequent, with moderate cases rarely exceeding 2% and severe cases (0.85%) observed only during the second to last assessment in building A. Sneezing and coughing were observed in every assessment, although their frequency varied (Table 2). Sneezing was more common early in the beginning of the fattening period, while coughing increased over time (Supplementary Figure 1). Both were highly correlated with weight—sneezing negatively, coughing positively (Figure 3). In none of the assessments and buildings these measures exceeded the warning threshold that impacts the “absence of disease” score. Laboured breathing (pumping) was only noted once in building A, exceeding the 1.8% warning threshold. Moderate hernias rarely exceeded 2%, and severe hernias were recorded in the fifth assessment in building C, surpassing the warning threshold of 0.6% (5). Skin conditions were scarce, rarely exceeding 1% (Table 2). Lastly, all pigs entered the fattening facilities with docked tails, a procedure carried out during earlier production stages without anaesthesia. No castration was performed.

Relating to the principle of “appropriate behaviour”, significant intra-farm variability was found for the fear of humans in three of the visits (Table 3). Values for QBA measures did not show extreme variation (Table 2), and several of them negatively correlated with weight: “active”, “tense”, “playful” and “distressed” states (Figure 3).

### 3.2 Criteria

**Figure 4:**
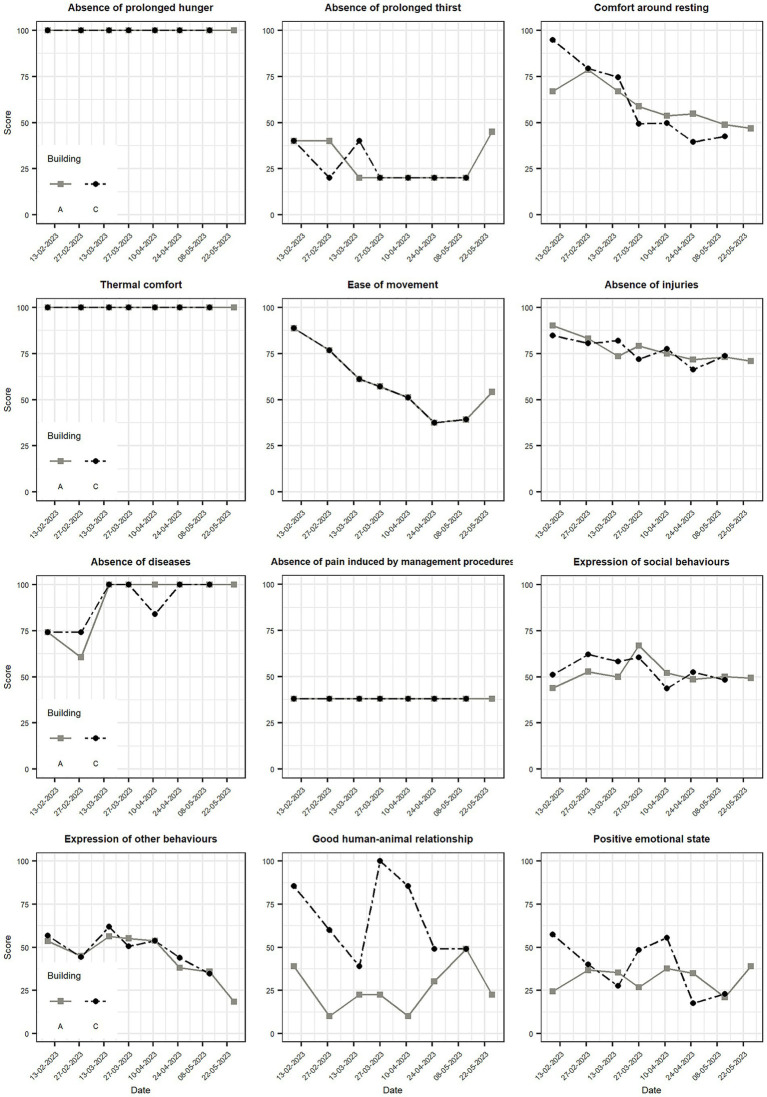
Progression over time of criteria scores from the Welfare Quality^®^ protocol for buildings A and C.

“Absence of prolonged hunger”, “thermal comfort” and “absence of pain induced by management procedures” criteria remained invariable during the study period (Table 4; Figure 4). Criteria scores were similar for both buildings except for “good human-animal relationship” and “positive emotional state” (Figure 4). The “expression of social behaviours” was similar for both buildings, with no evident trend, and had low variability over time as shown by the low standard deviation and IQR. On the other hand, the “expression of other behaviours” showed a downward trend. Two criteria under the “good housing” principle declined over time. The decrease in space allowance, caused by the pigs’ increasing body weight while pen size remained constant, led to the decay of the “ease of movement” criterion (Figure 4). A slight improvement was observed during the last two visits (excluding the final visit for building C), which can be attributed to a reduction in the number of animals in the evaluated pens. The “comfort around resting” criterion also showed a downward trend with one of the highest levels of variability.

**Table 4:** Descriptive statistics of the criteria scores from the Welfare Quality^®^ protocol for buildings A and C.

Criteria	Building A					Building C				
	Mean^1^	sd	Median	IQR	Range	Mean^1^	sd	Median	IQR	Range
1	100	0	100	0	[100, 100]	100	0	100	0	[100, 100]
2	28.12	11.32	20	20	[20, 45]	25.71	9.76	20	10	[20, 40]
3	59.41	10.72	56.8	14.45	[46.80, 78.50]	61.43	21.33	49.8	31.05	[39.50, 94.80]
4	100	0	100	0	[100, 100]	100	0	100	0	[100, 100]
5	58.25	17.49	55.7	16.9	[37.50, 88.70]	58.83	18.81	57.2	23.8	[37.50, 88.70]
6	77.1	6.67	74.25	7.43	[71, 90.20]	76.64	6.41	77.5	8.4	[66.30, 84.70]
7	91.84	15.54	100	6.47	[60.60, 100]	90.31	12.52	100	20.95	[74.10, 100]
8	38	0	38	0	[38, 38]	38	0	38	0	[38, 38]
9	51.74	6.73	50.05	3.08	[43.90, 67]	53.8	6.8	52.5	9.7	[43.60, 62.10]
10	44.56	13.2	49.3	16.52	[18.40, 56.40]	49.47	9.17	50.7	11.2	[34.70, 61.90]
11	25.75	13.42	22.5	13.02	[10.10, 49]	66.89	23.26	60.1	36.5	[39.10, 100]
12	32.02	6.86	35.2	10.8	[21, 39]	38.51	16.07	40.1	26.7	[17.50, 57.40]

Criteria: (1) Absence of prolonged hunger, (2) Absence of prolonged thirst, (3) Comfort around resting, (4) Thermal comfort, (5) Ease of movement, (6) Absence of injuries, (7) Absence of disease, (8) Absence of pain induced by management procedures, (9) Expression of social behaviours, (10) Expression of other behaviours, (11) Good human-animal relationship, (12) Positive emotional state. Criteria values range from 0 to 100. sd, standard deviation; IQR, interquartile range.

1 Means for each building were calculated as the average of visit-level means (Building A: *n* = 8 visits; Building C: *n* = 7 visits). Each visit-level mean was based on pens assessed during that visit according to the WQ^®^ protocol.

“Absence of disease” scores are obtained by warning and alarm thresholds (5). In the first two assessments, the mortality of the farm of 12 months was above the alarm threshold of 4.5% (5.14%), yielding a lower score for the criterion (Figure 4). The “absence of pain induced by management procedures” was constant since it represents management practices of farms that are not usually modified, certainly not during the fattening of a batch of pigs. The “absence of injuries” criterion showed a downward trend with a total reduction of 19.2 points for building A and 18.4 points for building C.

### 3.3 Principles

**Figure 5:**
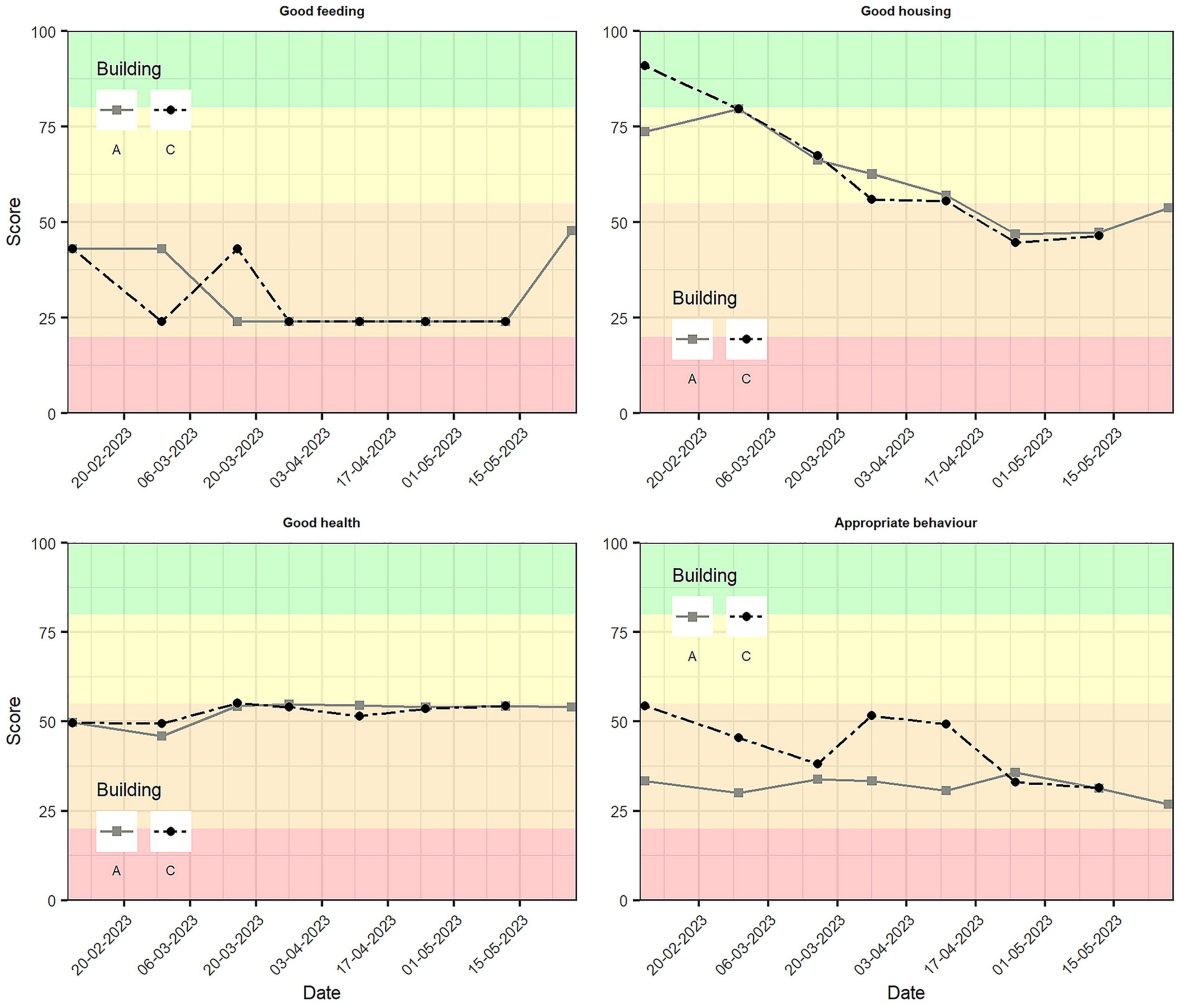
Progression over time of principle scores from the Welfare Quality^®^ protocol for buildings A and C. Welfare categories are shown: “Not classified” in red (0–20), “acceptable” in orange (>20–55), “enhanced” in yellow (>55–80), and “excellent” in green (>80–100).

The scores of principles fluctuated over time, affecting for some assessments their contribution to the welfare category. None of the principles scored in the “not classified” in any of the assessments (Table 5; Figure 5). “Good feeding” and “appropriate behaviour” fell in the acceptable range in all the assessments in both buildings. The scoring of the principle “good health” was repeatedly close to the threshold between “acceptable” and “enhanced”, and it was only categorized as “enhanced” in one assessment (third visit) of building C (Figure 5). Both “good feeding” and “good health” scores remained constant after the third visit for both buildings. As expected, “good housing” generally decreased in consecutive visits, and it was indeed the principle showing more variability over time. In the first assessment of building C, the score fell in the “excellent” category, being “enhanced” in the following four visits, and “acceptable” in the last two. Although “appropriate behaviour” scores fell always in the acceptable category, they were lower and more constant for building A, while they showed more variability for building C and a downward trend.

**Table 5:** Descriptive statistics of the principle scores from the Welfare Quality^®^ protocol for buildings A and C.

Principle	Building A					Building C				
	Mean	sd	Median	IQR	Range	Mean	sd	Median	IQR	Range
Good feeding	31.73	10.76	24	19	[24, 47.80]	29.43	9.27	24	9.5	[24, 43]
Good housing	60.89	11.91	59.8	16.05	[46.90, 79.60]	62.96	17.29	56	22.65	[44.70, 90.90]
Good health	52.73	3.2	54.2	1.4	[45.90, 54.90]	52.51	2.35	53.5	3.65	[49.40, 55.20]
Appropriate behaviour	31.9	2.79	32.35	3	[26.80, 35.70]	43.33	9.15	45.4	14.85	[31.50, 54.30]

Principle’s values range from 0 to 100. sd, standard deviation; IQR, interquartile range.

### 3.4 Overall welfare classification

The overall welfare classification slightly varied in building C, being categorized as “enhanced” in the third assessment, and the rest being “acceptable”. Building A was continuously categorized as “acceptable” in all assessments.

## 4 Discussion

The present study aimed to evaluate the temporal dynamics of animal welfare in a single batch of growing pigs distributed in two separate buildings by applying the WQ^®^ protocol across multiple time points. By tracking changes in measures, criteria, and principles throughout the fattening period, we explored how welfare scores evolved in response to animal growth, housing conditions, and management practices. Below, we examine the variability observed over time across the four WQ^®^ principles; “good feeding”, “good housing”, “good health”, and “appropriate behaviour”, and reflect on the implications of these findings for the robustness and reliability of welfare assessments in intensive pig farming systems.

### 4.1 Variability over time

Variability over time was found numerically and graphically in measures, criteria and, to a lesser extent, principles. As expected and previously described (6), the principles and criteria were more robust in terms of variability. This robustness was even more evident when applying the welfare categorization (5). These changes can impact the categorization and certification of farms in terms of animal welfare if not performed at the same fattening stage, which is precisely why certification is typically conducted at a standardized age, at the end of the production phase. For instance, the “good health” principle was categorized as “enhanced” in one out of seven assessments conducted in building C, which resulted in the overall category being changed to “enhanced” for that visit. Some studies assessed the variability of animal-based measures by the test–retest reliability for pigs in different life stages (10, 11, 13), and other species (6, 25). In pig studies, the test–retest reliability evidenced differences in some animal-based measures, but they did not evaluate the impact of variability in either the criteria or the principle scores (10, 11, 13).

#### 4.1.1 Good feeding

The “absence of prolonged hunger” criterion remained invariable throughout the study, suggesting that animals’ nutritional needs were consistently met, which is fundamental at this production stage. Currently, it is rare to find lean animals in production pens, as those in poor body condition are usually sick and thus moved to hospital pens, which are not assessed under the WQ^®^ protocol (5). In contrast, the “absence of prolonged thirst” criterion exhibited some variability. Across most visits, the number of available drinkers fell below the minimum thresholds recommended by the WQ^®^ protocol, which is 10 pig per drinker in the absence of liquid feeding (5), although Spanish legislation, through Royal Decree 159/2023, is less restrictive and establishes a minimum of one drinking point for every 12 pigs when dry feeding is used. In this case, the farm exceeded the maximum number of animals allowed per drinker; however, the legislation was in effect after the beginning of the fattening period, in March 2023, and it provided a two-year adjustment period for existing farms to comply with the new requirements. The observed fluctuations in this criterion are primarily attributable to the cleanliness and maintenance status of the drinkers rather than their absolute number. Dirty or poorly maintained drinkers likely reduced the effective availability of clean water, thereby negatively impacting this welfare indicator.

#### 4.1.2 Good housing

“Good housing” was the principle that showed greater variability in the current study, primarily due to declines in both “comfort around resting” and “ease of movement” criteria. The “ease of movement” decreasing trend is well explained by the presence of weight in the calculation of the “space allowance” measure (5). During fattening, pigs experience significant increase in size and weight gain leading to less effective space for animals to move if they remain in the same pen (26, 27). Thus, the age of the animals when the assessment takes place will highly influence the obtained score.

Reduced space allowance can influence animal behaviour by limiting avoidance capabilities, restricting the access to resources, and constraining resting areas, which can increase animal chronic stress and impair thermoregulation (26–28). In line with this, severe wounds became more frequent over time. However, this phenomenon was not accompanied by an increased in negative social behaviours during the assessments; rather the opposite trend was observed (Supplementary Figure 1). In addition, no evidence of tail biting was found, likely influenced by the practice of tail docking (29). Although activity is generally reduced with age (30), animals provided with more effective space remain more active and interact more with enrichment material (31–34). Similarly, reduced space allowance was associated with fouling on lying areas in partially slatted pens with higher pen density (35). This was reflected in our observations (Figure 3; Supplementary Figure 1), although the studied farm had fully slatted floors.

The decline in “comfort around resting” was linked to increased manure on the body and bursitis over time, both associated with greater body weight (16). Age-related increases in bursitis have been previously documented in intensively farmed pigs (36). Here, a significant positive correlation was evidenced between weight and both categories of bursitis, as well as weight and moderate manure. While Temple et al. described no significant effect of space allowance on the appearance of bursitis, they associated the increase of moderate bursitis with fully slated floors (36). Here, the prevalence of moderate bursitis was lower than in other studies conducted during the fattening period, whereas the prevalence of severe bursitis was comparatively higher (10, 16, 17, 36–38). The mean prevalence of severe bursitis on the farm (8.71%) can be comparable to the 10.8% described in fully slatted conventional farms (36). The prevalence of manure on the body was also in line with previous findings (11, 36). Seasonality was also attributed to this measure (11), since room temperature modifies the defecating behaviour of pigs (39, 40). Higher temperatures result in fouling of non-slatted floors and increasing wallowing behaviour, although wallowing is not exhibited in fully slatted floors (39, 40). However, the present study was conducted during a time of the year when environmental temperatures were not extreme and generally remained within ranges conducive to adequate thermoregulation in the animals. Although seasonality can affect several of the abovementioned measures, its influence could not be evaluated in this study.

#### 4.1.3 Good health

This principle was the most stable over time. Its score is more influenced by the “absence of disease” criterion, as this criterion is calculated based on warning and alarm thresholds (5). If values of the measures are below these thresholds, there is no impact on the criterion score. Low thresholds (warning below 2%) reflect the severity of some conditions, namely twisted snouts, pumping, rectal prolapse and severe hernias. Besides, animals suffering from these conditions should be relocated in the hospital pens, that are not visited during the execution of the WQ^®^ protocol (5). Hence, the presence of these pathologies above the threshold can be considered also as a management problem (17). Understanding how thresholds and the exclusion of hospital pens from evaluation influence scoring is essential for interpreting the impact of these measures on the “absence of disease” criterion and, consequently, on the “good health” principle. Among the respiratory manifestations assessed, sneezing and coughing were observed more frequently than other animal-based health measures. Sneezing also decreased over the fattening period in other studies (11, 41). The presence of both clinical signs cannot be necessarily associated with an infectious disease, since other factors such as poor air quality, lower temperatures, and ventilation issues can contribute to their appearance (42). Nevertheless, an outbreak of *Mycoplasma hyopneumoniae* was diagnosed in the studied farm during the monitored fattening period, as this bacterium was detected by PCR in tracheal secretions. *M. hyopneumoniae* is the primary etiological agent of the enzootic pneumonia and an important contributor to the porcine respiratory disease complex (43). This bacterium causes high morbidity and low mortality in affected herds, and clinical signs are characterized by dry, non-productive coughing and can have variable duration of up to months (44, 45). Although a respiratory pathogen was detected and laboured breathing exceeded the warning threshold during the second visit to building A, coughing frequency remained far below the warning threshold in all WQ^®^ assessments. The low observed coughing frequency may be explained by factors related to the host and pathogen (43), or by the relocation of severe cases to hospital pens (5), among others. This raises the question of whether the current threshold adequately reflects low to moderate and non-complicated respiratory disease presentations, as well as the impact of endemic diseases on animal welfare.

Within the “absence of injuries” criterion, moderate and severe lameness were detected at low prevalence. Similarly, other studies have reported comparable results in growing pigs (10, 11, 16, 17). The low prevalence can be attributed to methodological aspects of the assessment and relocation of animals in hospital pens (17). The prevalence of severe wounds never exceeded 4%, while moderate wounds on the body showed a positive trend and was more frequent. As previously mentioned, aggressions and wounds on the body can increase due to the reduction of space allowance (26, 28). Besides, regrouping is associated with hierarchical fights, which happens at the beginning of the fattening period (46). In the present study, the lowest prevalence of wounds was recorded in both buildings during the first WQ^®^ assessment, but the highest negative social behaviour was observed.

#### 4.1.4 Appropriate behaviour

The “expression of social behaviours” criterion was fairly stable. The “social behaviour” measure had a negative trend, but the range of values was low. The overall prevalence was similar to prevalences observed in other studies directed at fattening pigs (10, 11, 16, 17, 38). This measure could decrease due to the reduction of space allowance (26). In fact, “social behaviour” and the term “active” emotional state from the QBA were positively correlated.

The “expression of other behaviours” is composed by the exploratory behaviour of pigs including elements of the pen and enrichment material. Exploration is a key natural behaviour of pigs (47), yet a downward trend was observed during the final three visits, contrasting with previous findings (11). Space allowance and increasing age can both limit exploratory behaviour and interaction between pen mates (31–33). Remarkably, straw was provided as environmental enrichment in this farm; a well-studied materials deemed adequate to express the rooting behaviour since it is manipulable, chewable and destructible (47). However, it was unclear whether straw was provided consistently throughout the fattening period, and continuous provision is essential to sustain exploration levels (31, 47).

The “fear of humans” measure was very variable and inconsistent during the studied period. Our findings contrast with Czycholl et al. that described a decrease in panic response with the increase of age as pigs get more used to human handling. They also found a low test–retest reliability in similar age groups (11). In contrast, good test–retest reliability was obtained with a year-interval (10).

The QBA evaluates the emotional state of the animals, which is a cornerstone of animal welfare (48). Some significant correlations were found between emotional states as well as the time-dependent weight, showing an evolution of the emotional state of animals during the fattening period. Broadly, pigs were less active, tense, distressed and playful (Figure 3). Nevertheless, the overall variability was moderate.

### 4.2 Intra-farm variability

One of the focuses of this study was the evaluation of the intra-farm variability. Temple et al. pointed out that conditions with lower prevalence in the herd yielded high intra-farm variability considering the pen as the analysis unit (17). In this case, some conditions were observed in singular assessments in a specific building, namely severe lameness and pumping in building A, and severe hernias in building C. However, for the final categorization as well as certification purposes, variability of measures is not as important as variability of principles, which determine the outcome of the farm in terms of animal welfare (5).

Significant differences between buildings were found in certain assessments for “fear of humans”, absence of bursitis, moderate bursitis, absence of manure, and moderate wounds. These results show the variability of buildings in the same farm. The “fear of humans” was the most variable, and generally more prevalent in building A (Supplementary Figure 1). Doubts about the uncertainty, reliability, and validity of the measure of human-animal relationship (HAR) has been risen, since the trigger of a panic response in the animals is multifactorial and susceptible to minor changes (8). HAR measure is likely shaped not only by pigs’ experiences and genetics, but also by daily handling, their environment, and the manner in which the assessment is conducted (18, 49). Handling techniques and caretaker behaviour towards pigs are recognized as key factors influencing HAR (49, 50). In our study, these factors were consistent across buildings because the same caretakers handled both groups, and management practices were uniform across batches under an all-in/all-out system. Therefore, systematic differences related to handler identity, experience or routine interactions are unlikely to explain the observed variability. Nevertheless, variation in the fear-of-humans measure may still arise from additional factors affecting pig responsiveness, including individual behavioural traits (48, 51), differences in daily handling intensity, and transient noise or activity in the surroundings. These factors may contribute to between-building differences despite overall consistency in routine management.

### 4.3 Limitations and future directions

Despite using the same trained assessors to ensure consistent inter-observer variability across visits, previous studies have reported varying levels of inter-observer reliability in the WQ^®^ protocol, particularly for specific measures such as bursitis (37), QBA (12), manure on the body, and lameness (52). The evaluation of inter-observer reliability was not feasible, as the assessors did not observe the same animals. In any case, the main limitation of this study is the low number of units of analysis, that hampers the application of certain methodological approaches to evaluate reliability (9, 10), and limits the statistical power (53). Limitations in statistical power make more probable that some true effects remain missing, and that an overestimation of correlation coefficients could be present (53). Therefore, conclusions should be considered descriptive and interpreted alongside existing literature. Further studies with an increased sample size, and ideally including different fattening systems from different countries, need to be carried out to draught more solid conclusions about the consistency over time and intra-farm variability of animal welfare indicators over the fattening of growing pigs.

## 5 Conclusion

Animal welfare is increasingly valued by consumers, making the perceived reliability and robustness of welfare certification processes critically important. This study revealed variability in some measures, criteria, and principles of the WQ^®^ protocol throughout a single fattening period. These fluctuations, particularly at the principle level, highlight the potential impact of assessment timing on animal welfare outcomes, stressing the importance of certification performance on the last weeks of the fattening cycle. Altogether, the present study underscores the need for further research to evaluate how temporal and intra-farm variability may affect the consistency and credibility of animal welfare assessments, and to explore strategies to enhance the reliability of existing protocols under dynamic production conditions.

## Data Availability

The raw data supporting the conclusions of this article will be made available by the authors, without undue reservation.
