# Supplementary material for: Temporal dynamics and intra-farm variability of animal welfare indicators in fattening pigs
Source: Front Vet Sci. 2026 Mar 30;13:1797211. doi: 10.3389/fvets.2026.1797211 (PMC13073092; doi:10.3389/fvets.2026.1797211)
Supplement: Supplementary file 1 [file Image_1.pdf]

Supplementary Material

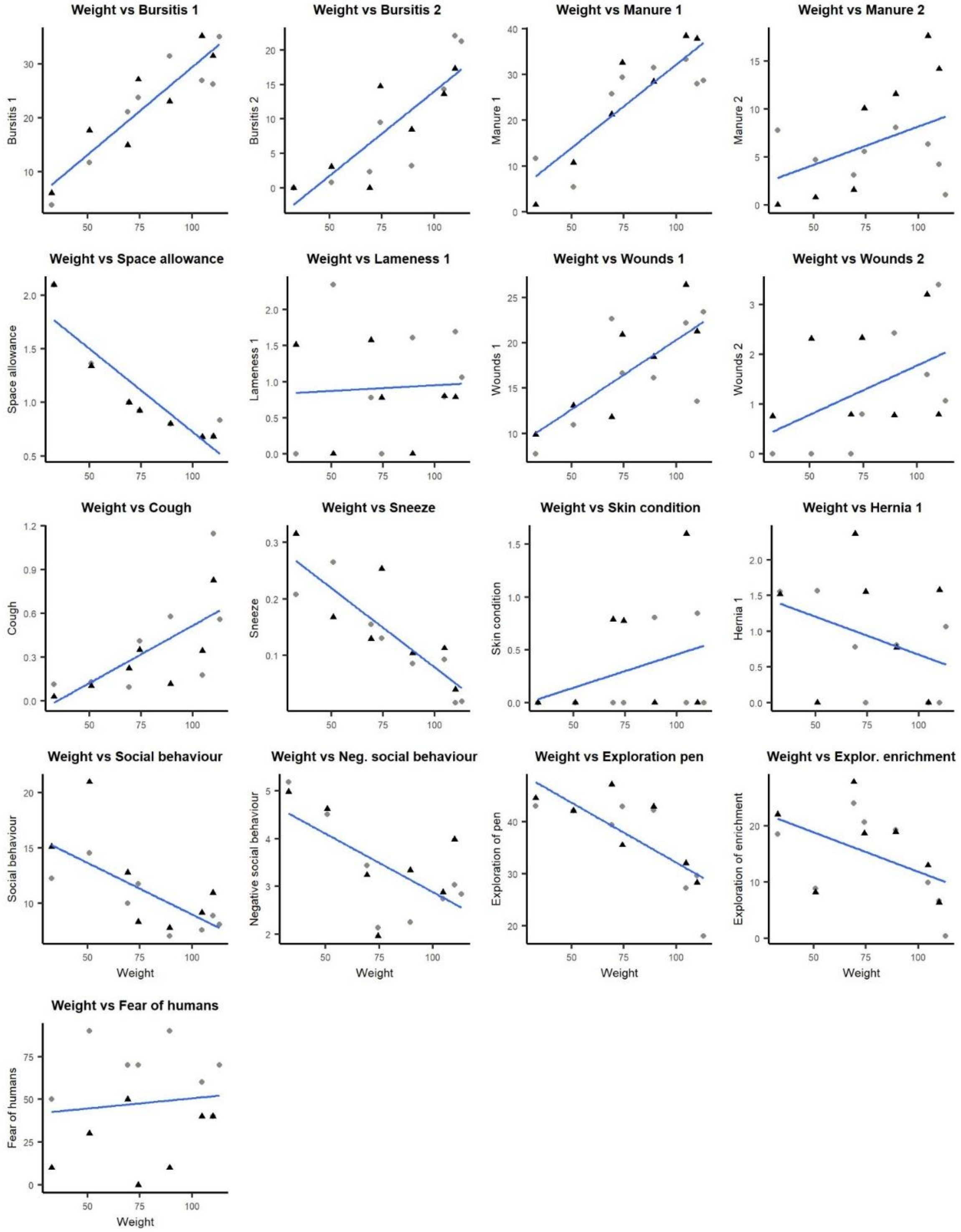

Supplementary Figure 1. Relationship between measures with some variability and weight. The number in some measures corresponds to the category of the severity of the condition: 1 = moderate, and 2 = severe. Building A is represented by a grey circle (●), while building C is represented by black triangles (▲).

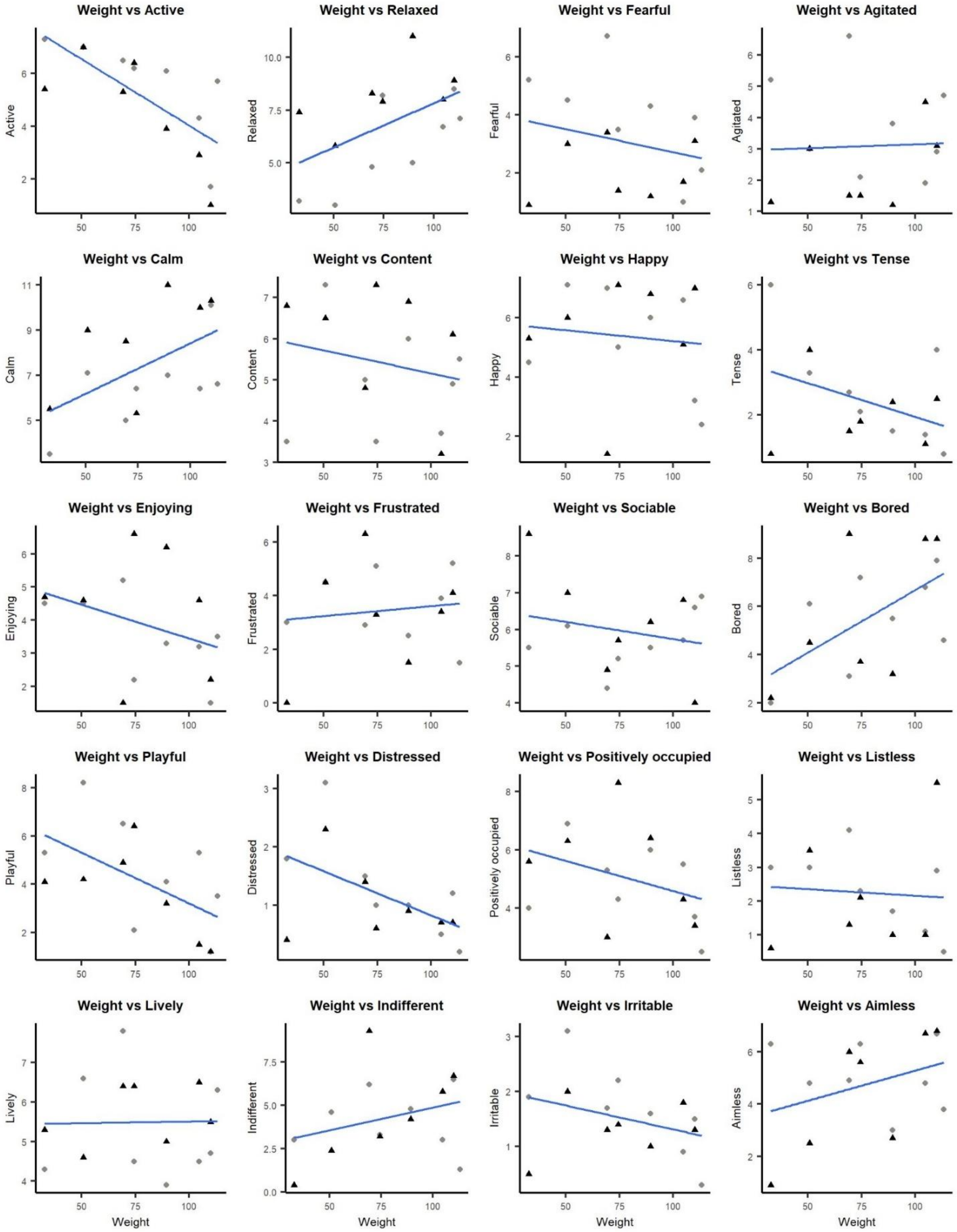

Supplementary Figure 1 (cont.). Correlation plots between measures with enough variability and weight. Building A is represented by a grey circle (●), while building C is represented by black triangles (▲).
